# Additively Manufactured Continuous Cell-Size Gradient Porous Scaffolds: Pore Characteristics, Mechanical Properties and Biological Responses In Vitro

**DOI:** 10.3390/ma13112589

**Published:** 2020-06-05

**Authors:** Fei Liu, Qichun Ran, Miao Zhao, Tao Zhang, David Z. Zhang, Zuqiang Su

**Affiliations:** 1 School of Advanced Manufacturing Engineering, Chongqing University of Posts and Telecommunications, Chongqing 400065, China; liufei@cqupt.edu.cn (F.L.); suzq@cqupt.edu.cn (Z.S.); 2 College of Communication and Information Engineering, Chongqing University of Posts and Telecommunications, Chongqing 400065, China; 3 State Key Laboratory of Mechanical Transmission, Chongqing University, Chongqing 400044, China; zhaomiao@cqu.edu.cn (M.Z.); zghzt@hotmail.com (T.Z.); 4 College of Engineering, Mathematics and Physical Sciences, University of Exeter, North Park Road, Exeter EX4 4QF, UK; zhangzw@cqu.edu.cn

**Keywords:** additive manufacturing, selective laser melting, graded porous scaffold, triply periodic minimal surfaces, mechanical behavior, pore characteristics

## Abstract

Porous scaffolds with graded open porosity combining a morphology similar to that of bone with mechanical and biological properties are becoming an attractive candidate for bone grafts. In this work, scaffolds with a continuous cell-size gradient were studied from the aspects of pore properties, mechanical properties and bio-functional properties. Using a mathematical method named triply periodic minimal surfaces (TPMS), uniform and graded scaffolds with Gyroid and Diamond units were manufactured by selective laser melting (SLM) with Ti-6Al-4V, followed by micro-computer tomography (CT) reconstruction, mechanical testing and in vitro evaluation. It was found that gradient scaffolds were preferably replicated by SLM with continuous graded changes in surface area and pore size, but their pore size should be designed to be ≥ 450 μm to ensure good interconnectivity. Both the Gyroid and Diamond structures have superior strength compared to cancellous bones, and their elastic modulus is comparable to the bones. In comparison, Gyroid exhibits better performances than Diamond in terms of the elastic modulus, ultimate strength and ductility. In vitro cell culture experiments show that the gradients provide an ideal growth environment for osteoblast growth in which cells survive well and distribute uniformly due to biocompatibility of the Ti-6Al-4V material, interconnectivity and suitable pore size.

## 1. Introduction

Porous Ti6Al4V scaffolds with excellent biocompatibility and interconnected and complex porous architecture have been considered as promising orthopedic implants owing to their low elastic modulus matching that of natural bone and biological performances that can induce tissue ingrowth [1,2]. The scaffolds provide adequate space for capillaries formation and bony tissue ingrowth for better fixation with host tissue [3,4]. Recently, metallic additive manufacturing (AM) technologies, such as electron beam melting (EBM) [5,6] and selective laser melting (SLM) [7,8] have been successfully applied to build porous scaffolds with precisely controlled pore shapes and interconnectivities suitable for facilitating tissue ingrowth, which has received extensive attention. These scaffolds with high porosity provide multiple advantages, including suitable mechanical properties [9] and abundant interconnected pores, which benefit body fluid circulation, nutrient and oxygen transport and vascularization [10]. Having microstructural and mechanical properties similar to natural bones enable the porous scaffolds to exhibit good permeability and mechano-transduction, which has a significant effect on tissue regeneration efficiency [11,12].

Additively manufactured porous scaffolds have been developed and widely investigated in implants of the orthopedic field [3,4], demonstrating that the scaffolds with morphological properties similar to that of natural bone can promote tissue ingrowth. However, due to the complexity of the architecture and the variability of properties within natural bone, the current challenge is to locally manipulate the internal porosity and pore shape to meet the requirements of specific repair sites [1,13], for instance, native tissues consisting of inhomogeneous structures [14,15]. Cancellous bone with a large number of spongy trabeculae provides good interconnectivity for metabolic activity and allows room for blood vessel and marrow penetration. In contrast, cortical bone, which is much denser than cancellous bone, plays a supporting role, protects body’s organs and provides a moving lever [16].

In this regard, it is necessary to develop scaffolds with continuous functional gradients similar to that of the host tissue, meeting the demands of various morphological properties to better play the part of the extracellular matrix. Gradient features that combine the mechanical and biological properties with graded architectures could promote the formation of multiple tissues and tissue interfaces [11,17], resulting in a more robust tissue–material interfacial bonding strength.

Recently, implicit surface modeling (ISM) with a compact mathematical representation has become a promising design approach for the generation of cellular structures [2]. Using the ISM approach, a series of triply periodic minimal surface (TPMS) unit cells [18,19], such as Primitive, Diamond and Gyroid, have been modeled with biomorphic scaffold architectures. Melchels et al. [20] found Gyroid scaffolds could improve cell seedability when compared with the salt-leached scaffolds, facilitating a homogeneous cell distribution with sufficient nutrient and oxygen supply. Graded cellular structures with Diamond [21] and Gyroid [22] units have been readily generated by the ISM method to tailor the mechanical properties. Maskery et al. [23] designed hybrid cellular structures using the sigmoid function, proposing a novel solution to a structural weakness problem between their interfaces [23]. Furthermore, TPMS structures provide a specific performance, i.e., having a mean curvature of zero, that coincides with that of trabecular bone [24,25] and other native tissues of a variety of species [19]. Fabricating TPMS structures by SLM and performing an experimental test, Yan et al. [9] found that these structures provide a porosity comparable and a modulus similar to those of natural bone. Two TPMS cellular structures with skeletal and sheet types were studied by Al-Ketan et al. [26], uncovering that their mechanical properties are higher than that of strut-based structures. Additionally, low anisotropy of the Gyroid structures was found by Yang [27], who investigated the orientation dependence of the mechanical responses. Moreover, compared to the polyhedral units with beam-like struts and sharp turns, TPMS architectures feature continuous and optimized pore shapes that are conducive to reduce stress concentration under loading, leading to a longer fatigue life [19]. Gradient scaffolds in multiple patterns, such as density gradients, heterogeneous gradients and cell-size gradients, can be constructed to better mimic the morphologies of host tissue [28].

Meanwhile, many studies have been conducted to investigate porous scaffolds with polyhedral units in terms of their biological behaviors. For example, Taniguchi et al. [4] investigated Diamond lattice scaffolds implanted into rabbit tibia and found that metallic scaffolds with pore sizes of 600 μm exhibit a significantly higher fixation ability for orthopedic implants than those with pore sizes of 300 and 900 μm. In general, the pore size is recommended to be greater than 300 μm to enhance new tissue and capillary regeneration [1]. In vivo, it has been suggested that porous titanium implants should have a pore size of 490–1100 μm, which exhibits a reasonable property for the growth of biological tissue [29,30]. To date, it is acknowledged that physicochemical properties, such as the porosity, pore shape, pore size, interconnectivity, and surface wettability, more or less determine the bone-implant osteointegration capacity [31].

However, graded structures of TPMS, as a promising biological alternative for implants, are lacking of pore characteristics to validate their performances. Far fewer investigations have been conducted on biological behaviors of cell-size gradient scaffolds fabricated by SLM. Therefore, it is of great significance to gain insight into their biological responses. On the other hand, this graded change in cell size will also impact the mechanical properties, which needs to be studied.

Towards that end, this study is devoted to evaluating SLM scaffolds with a continuous cell size gradient in terms of their pore characteristics, mechanical properties and bio-functional performances. Cell-size gradient scaffolds ranging from 0.6 to 2 mm (the corresponding pore size is approximately 200 to 1000 μm) with Gyroid and Diamond units were designed by the ISM method and used for in vitro experiments to evaluate their ability to support bone cell growth. Meanwhile, the same structures with graded and uniform unit cells were subjected to compressive tests to detect their mechanical behaviors. All of their relative density was fixed in 30% for a suitable mechanical property. Prior to this, all of those samples were fabricated by SLM technology with Ti6Al4V powder, followed by micro-computer tomography (CT) reconstruction for accuracy confirmation and to visualize the pore shape.

## 2. Materials and Methods

### 2.1. Design Approach of Scaffolds and Manufacturing

**Figure 1:**
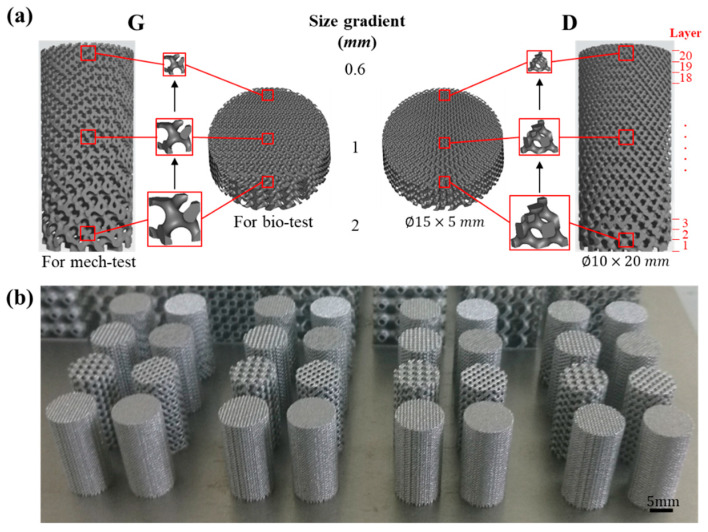
(**a**) Designed models of cell-size gradient samples and (**b**) as-built samples for compressive tests.

The porous scaffolds studied in this work were designed using the ISM method according to our previous study [28]. Two types of porous units, i.e., Gyroid (G) and Diamond (D), were constructed in MATLAB software (R2014a, MathWorks, Natick, MA, USA), and their program codes are shown in Supplementary Materials. Then Magics software (20.03, Materialse, Leuven, Belgium) was used to scale and refine the CAD files. All of the samples studied in this work fall into two categories based on their experimental use, one for mechanical performance tests with dimensions of *ϕ*10 × 20 mm and the other one for biological experiments in vitro with dimensions of *ϕ*15 × 5 mm, as shown in Figure 1a. Figure 1b shows a portion of the as-built samples fabricated by SLM.

As mentioned above, the tissue growth activity is excellent in vivo when the porous titanium implant has a pore size of 490–1100 μm [29,30]. According to measurements in Magics software, the cell sizes of the Gyroid and Diamond structures corresponding to this range are approximately 0.6 to 2 mm. Therefore, the cell size of the gradient structure was designed in this range, for which the sample is marked as ‘Graded 0.6–2′ for the both of Gyroid and Diamond, as listed in Table 1.

Meanwhile, to obtain a comprehensive overview of the mechanical properties of different cell size structures, uniform porous structures with cell sizes of 0.6, 1, and 2 mm were generated and marked as ‘G 0.6′, ‘G 1′, ‘G 2′ for Gyroid (‘D 0.6′, ‘D 1′, ‘D 2′ for Diamond). In addition, ‘G vitro’ and ‘D vitro’ denote the gradient samples used for the biological experiments, with the same gradient change as mechanical ones. Table 1 provides detailed information about the samples used for experiments.

**Figure 2:**
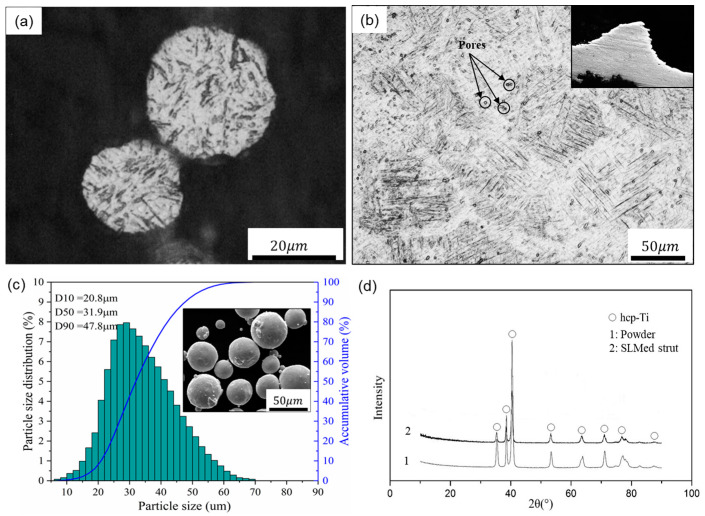
Optical micrographs of (**a**) the powder and (**b**) a strut cross section, (**c**) particle size distribution and SEM image of the Ti-6Al-4V powder, and (**d**) XRD patterns of the powder and SLM produced strut.

A SLM machine (EOSINT-M280, EOS GmbH, Munich, Germany) equipped with a 200 W CW Ytterbium fiber laser was used to fabricate the porous scaffolds. The processing parameters and manufacturing procedures were shown in the supplementary document accompanying the paper (Table S1 in Supplementary Materials). When the SLM process was finished, the porous samples were removed from the base plate and annealed at 650 °C for 2 h in argon to eliminate thermal stress. In addition, Ti-6Al-4V powder, characterized by corrosion resistance, good biocompatibility and excellent mechanical properties, was used to produce the porous scaffolds. It has a nearly spherical shape, smooth surfaces and a narrow particle size distribution in the range of 20–53 μm, providing a guarantee for precision shaping, as shown in the following Figure 2c.

### 2.2. Porous Biomaterial Morphology

Before the morphological and mechanical characterizations, samples were cleaned by ultrasonic machine in demineralized water for 20 min to remove the residues. A micro-computer tomography (CT) scanner (diondo d2, Hattingen, Germany) with an isotropic voxel size of 6.45 μm was used to scan cell-size gradients at 210 kV voltage and 110 μA current, generating two-dimensional (2D) slice image data. During the scanning processing, the samples were rotated over 360° in steps of 0.2°. The total acquisition time was about 80 min for each sample. Then the reconstruction 3D models were established and visualized through the 2D images using Mimics17.0 software (Materialise, Leuven, Belgium).

Based on the 3D models, the surface area and volume of the Gyroid and Diamond gradients with dimension of *ϕ*10 × 20 mm were measured using Magics software and compared with their respective design counterparts. For simplification of the statistics, each model was evenly divided into 20 parts, marked “Layer 1–20” from bottom to top, as shown in Figure 1a. Specifically, as in Reference [32], the pore dimensions were measured in a 2D slice image of the corresponding layer by Mimics, in which pore size value was obtained by inscribed circles of five random pores. The obtained data of design model and CT model include surface area (S), volume (V) and pore size (P), which were performed by Origin 2018 software (OriginLab, Wellesley Hills, MA, USA) for statistical analysis, as shown in Table S2 (Gyroid gradient) and Table S3 (Diamond gradient) in Supplementary Materials.

### 2.3. Mechanical Testing

Porous samples with dimensions of *ϕ*10 × 20 mm were subjected to uniaxial compressive tests using an MTS servo-hydraulic machine (MTS Systems Co., Ltd., Shenzhen, China) with a 100 kN load cell. The loading rate was set at a cross-head speed of 1.5 mm/min, recorded by a digital camera to keep track of their deformation behaviors. Three sets of repeated experiments were performed under the same normal condition according to the ISO 13314:2011 standard [33]. In this study, elastic modulus (*E*) was defined as the slope of stress–strain curve within the elastic deformation region, yield strength (*σ*_s_) was measured as the compressive 0.2% offset stress, and ultimate strength or collapse strength (*σ*_b_) was determined as the stress at the first peak on the stress-strain curve.

### 2.4. In Vitro Study

#### 2.4.1. Cell Culture

Primary osteoblasts were cultured according to a previous work [34]. In brief, neonatal Sprague-Dawley rats were sacrificed and disinfected in 75% ethanol and distilled water, then the calvaria was extracted and gently rinsed with sterilized phosphate buffer saline (PBS). The calvaria was cut into pieces and placed in a cell culture flask for 12 h. Then the fragments were incubated in Dulbecco’s modified eagle medium (DMEM) with high glucose, supplementing with 10% fetal bovine serum (FBS) and 1% penicillin/streptomycin. Incubation of the cells was carried out in a humidified atmosphere of 5% CO_2_ at 37 °C, followed by a refreshment of cell culture medium every other day. When 80% confluence was reached, the third generation of osteoblasts was used for subsequent experiments.

#### 2.4.2. Cell Adhesion and Morphology

In this work, osteoblasts were seeded at a density of 5 × 10^4^ cells/cm^2^ onto the substrates which were placed in 24-well culture plates. After being cultured for 2 days, the samples were fixed with 4% paraformaldehyde and gently rinsed with PBS. For SEM observation, samples were dehydrated several times in gradient ethanol solutions, followed by dried treatment with t-butanol for 30 min. Cell morphology was observed by field emission scanning electron microscopy (FE-SEM; JSM-7800F, JEOL, Akishima, Japan). Then for cell fluorescence observation, the samples were permeabilized with 0.2% Triton X-100. Then Rhodamine-Phalloidin and Hoechst 33258 were used for staining of cell cytoskeleton and cell nuclei, respectively. Finally, fluorescence microscopy (Leica DMI 600B, Wetzlar, Germany) was employed to detect the morphology and distribution of cells.

#### 2.4.3. Cell Viability and Proliferation

In this work, osteoblasts were seeded at a density of 5 × 10^4^ cells/cm^2^ onto the substrates which were placed in 24-well culture plates. After 4 days of incubation, the cells were stained with fluorescein diacetate (FDA) to evaluate their viability using fluorescence microscopy (Leica AF6000, Wetzlar, Germany). Before observation, the cultured samples were gently washed 3 times with PBS; and 10 μL of FDA solution (5 mg/mL) was added to each well and incubated at 37 °C for 10 min.

To further identify the cell proliferation, the substrates were gently washed with PBS, and cultured in DMEM with 10% thiazolyl blue tetrazolium bromide (MTT) in a 5% CO_2_ atmosphere at 37 °C for 4 h. Afterwards the medium was discarded, 500 μL of dimethyl sulfoxide (DMSO) was added to dissolve the resulting violet crystals. Finally, a spectrophotometric microplate reader (Bio-Rad 680, Hercules, CA, USA) was applied to measure the optical density (OD) at a wavelength of 490 nm.

#### 2.4.4. Alkaline Phosphatase (ALP) Measurement

After incubation for 7 days, the cells were lysed with 1% Triton X-100 at room temperature for 30 min. The lysate was mixed with alkaline phosphatase and incubated in a 37 °C environment for 15 min. Similarly, the spectrophotometric microplate reader (Bio-Rad 680, Hercules, CA, USA) was applied to measure the optical density (OD) at a wavelength of 520 nm. A bicinchoninic acid (BCA) assay kit (Beyotime Biotechnology Co., Haimen, China) was used to determine total protein content, followed by normalization.

Cell lysates were collected to determine total intracellular protein and ALP activity with bicinchoninic acid (BCA) assay kit.

## 3. Results and Discussion

### 3.1. Microstructure and Morphology of the Porous Biomaterials

According to a surgical implant standard (ISO 20160:2006), titanium implant materials need to have a homogeneous equiaxial microstructure, providing material integrity and good mechanical properties [35]. In this regard, Figure 2a,b shows the microstructure of the Ti-6Al-4V powder and SLM-produced strut with optical micrographs, demonstrating that the SLM parts have near fully dense struts except for a very small amount of spherical or irregular pores. In addition, the SLM strut exhibits columnar grains filled with very fine α’ martensitic laths that are oriented orthogonally. This result is obtained due to the high-speed heating and cooling rates effects of the SLM process, forming fine microstructures during the rapid solidification and subsequent martensite transformation, resulting in columnar grains [36,37].

Additionally, Figure 2d depicts the XRD patterns of the Ti-6Al-4V powder and SLM produced strut, and only peaks corresponding to the hexagonal close-packed titanium (hcp-Ti) phase were detected within them. These hexagonal close-packed phases include α-Ti and α’ martensite, and they have the same crystal structure and similar lattice constants. Compared with the powder state, the intensity of the α’ phase of the SLM-produced strut is significantly reduced and the diffraction peak is relatively broad, indicating that the SLM-produced part forms finer crystals and microstructure, meeting the requirements of implant materials.

**Figure 3:**
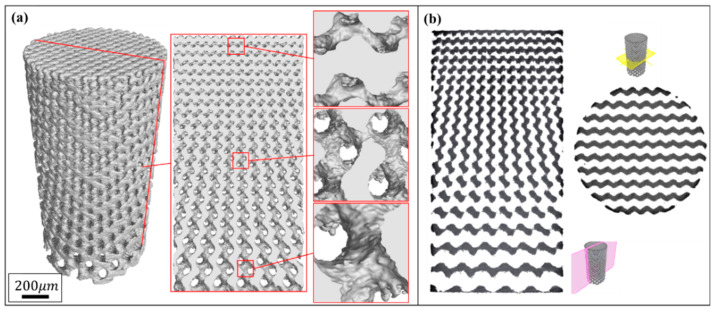
(**a**) Micro-computer tomography CT) reconstruction models of Gyroid cell-size gradient samples with different magnifications, and (**b**) corresponding 2D slice images.

**Figure 4:**
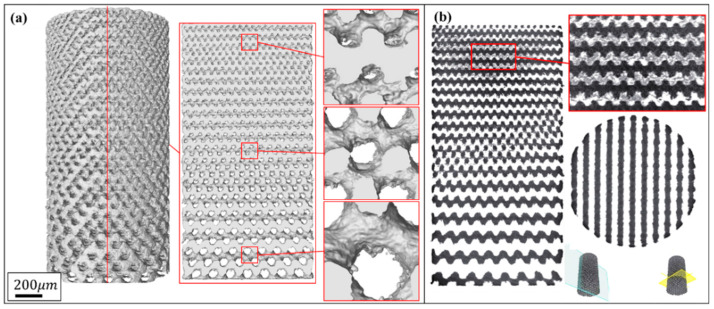
(**a**) Micro-CT reconstruction models of Diamond cell-size gradient samples with different magnifications, and (**b**) corresponding 2D slice images.

Figure 3 and Figure 4 show the characterization of Gyroid and Diamond cell-size gradient samples, respectively, reconstructed by micro-CT. A good interconnectivity and well-controlled pore shape can be observed within the porous scaffolds, demonstrating a good reproducibility of the SLM parts relative to the originally designed ones. The cross-sectional profile provides a uniform pore size distribution at a specific height, which is similar to the designed model. In contrast, the vertical profile shows that the pore size gradually decreases in the height direction, accompanied by thinner struts and more pores from bottom to top. However, the magnified view reveals that the surface of the structure is rough. The manufacturing precision and roughness of the SLM parts are determined by factors such as laser scanning accuracy and particle size of the powder. Thus, the roughness is basically the same throughout the structure, resulting in a shape of the microstructure poorly similar to original model. Fortunately, no closed pores or no broken struts were noticed, indicating that the use of SLM process to manufacture porous structures has good reliability.

#### 3.1.1. Surface Area

**Figure 5:**
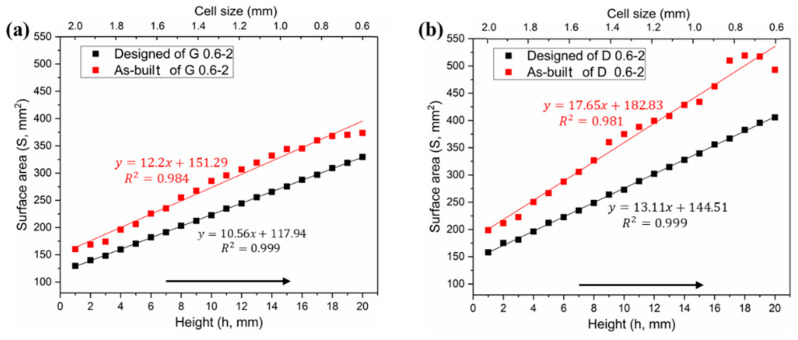
Comparative analysis of surface area distributions between designed and as-built models, (**a**) Gyroid gradient porous structure, and (**b**) Diamond gradient porous structure.

**Figure 6:**
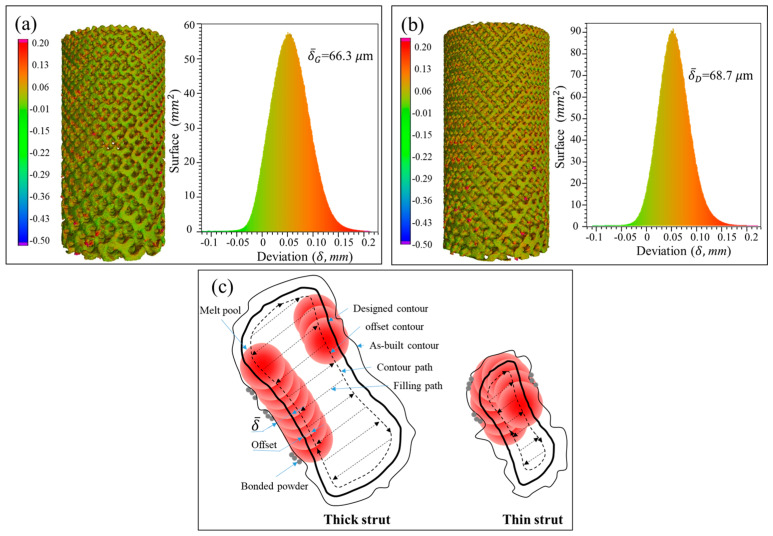
Statistical analysis of the deviation between designed and as-built parts for G (**a**) and D (**b**) structure, and (**c**) schematic diagram for the deviation of as-built parts.

As a result of cell-size gradient design, the surface area and pore size change along the Z-axis, as shown in reconstructed micro-CT models. The surface areas of both designed and CT models increase linearly with increasing height, giving two sets of approximately parallel lines in Figure 5a,b. Due to the influence of the corrugations and roughness in the SLM process, a certain difference value between designed and as-built model should have been expected for both of Gyroid and Diamond structures, displaying completely parallel lines. However, a divergent line has been seen in Figure 5b, which may be attributed to the larger surface and thinner struts at the top of Diamond gradient. As the height grows, the scanning contour line also increases, leading to the laser spot in the thin struts overlapping and thus accumulating more energy density, resulting in an increase of partially melted powder and rough surface (see Figure 6c).

According to the measured data in Tables S2 and S3 in Supplementary Materials, the Gyroid CT model has a higher surface area than the designed one by an average of 22.42% ± 3.79%, while the Diamond CT model provides an additional value of 29.97% ± 5.1%. Figure 6a,b shows the deviation distribution of the as-built G and D structures, respectively, illustrating both visual and quantifiable comparisons to their designed CAD models. In agreement with the results obtained in article [38], the deviations are lower than 0.2 mm over most areas of the entire parts, indicating a high accuracy of cell-size gradients fabricated by SLM.

In the SLM process, the laser scanning includes center filling and contour path generation according to the part geometry, as shown in Figure 6b from G struts. Owing to the influence of the laser spot with Gaussian heat source, it forms a heat affected zone of a certain width during scanning, which enables the powder at the contour boundary to be partially melted and resultantly attached to the solid interface [39]. Some powders are also adsorbed by the molten pool, solidified and bonded to the surface of the final part. It is these irregular morphologies and roughness of strut surfaces that increase the surface area and volume of the original designed part [40].

#### 3.1.2. Volume and Relative Density

**Figure 7:**
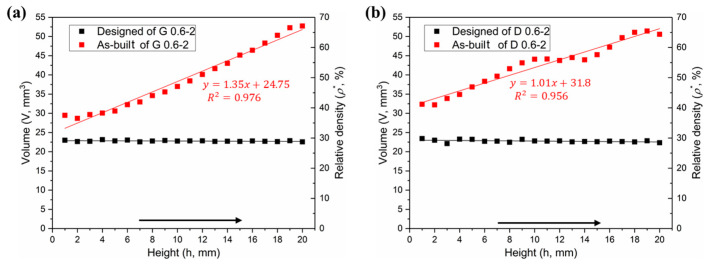
Comparative analysis of volume distributions between designed and as-built models, (**a**) Gyroid gradient porous structure, and (**b**) Diamond gradient porous structure.

In theory, the cell-size gradients studied in this work have a uniform relative density in all layers, which was confirmed by the two horizontal lines in Figure 7a,b. However, the volume in the as-built model shows a linear increase in the height direction, as similar to the surface area. It has been also found in a study [38] that the relative density and volume fractions of the SLM cellular parts are higher than those designed ones, due to the increase of the strut and the bonded powder particles.

Figure 6a,b provides the statistical analysis of the deviation between the designed and as-built parts of the G and D structure, respectively, and Figure 6c gives the reason for the deviation. The linearly increasing volume is associated with the surface area of the porous structure. To find the relationship between them, the following formula was established:

(1) $$\delta =\Delta V/S_{0}$$

where Δ*V* = *V*_1_ − *V*_0_, i.e., the difference of volume between the as-built and designed models. *S*_0_ represents the surface area of designed model, and *δ* is the nominal thickness of increased volume. The *δ* values for each layer of the Gyroid and Diamond structures are listed in Tables S1 and S2 of Supplementary Materials, respectively. It was found that *δ*_G_ and *δ*_D_ are very close, calculated as *δ*_G_ = 66.3 ± 17.1 μm, *δ*_D_ = 68.7 ± 6.8 μm, giving no significant deviation; accordingly, the total average is *δ* = 67.6 ± 12.9 μm, which can used to predict the geometric characteristics of the porous scaffolds for future application of biological transplantation. Thus, this invariable thickness is multiplied by the surface area that linearly increases along the Z-axis, resulting in a linearly increased volume, as shown in Figure 7a,b.

#### 3.1.3. Pore Size

**Figure 8:**
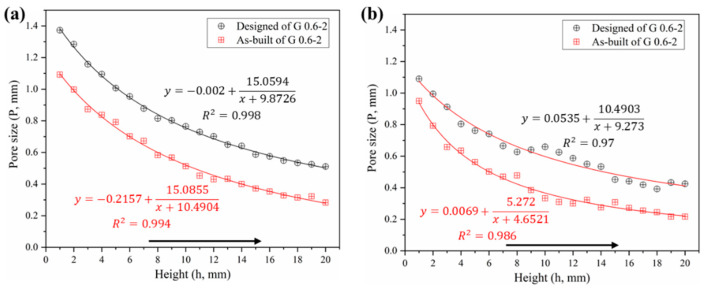
Comparative analysis of pore size distribution between designed and as-built models, (**a**) Gyroid gradient porous structure, (**b**) Diamond gradient porous structure.

The pore size is another issue of concern for bone grafts. Figure 8 shows the comparison of the pore size distributions among both designed and CT models. In general, one can note that the pore size of the CT models is substantially smaller than the designed ones, which validates the conclusion of the increased volume mentioned above. Specifically, the average pore sizes of Gyroid and Diamond were reduced by 241 ± 27.7 and 213 ± 56.4 μm, respectively. These two values turned out to be similar, providing a total average of 227 ± 46.1 μm, larger than the double thickness (2*δ* = 135.2 μm) due to the rough surface. Theoretically, if the surface is completely smooth, the difference value (Δ*P*) between the designed and as-built pore size should have been Δ*P* = 2*δ*. However, due to the irregular morphologies and roughness of the strut surfaces, the pore size has been further reduced, resulting in Δ*P* − 2*δ* = 91.8 μm, which should be paid attention to in future designs to ensure that the resulting structure has fully interconnected porosity.

In contrast, the pore size of the Diamond structure is smaller than that of the Gyroid counterpart at the same height position. Their differential average is 142 ± 56 μm in as-built models according to Tables S2 and S3 in Supplementary Materials. Some of the powders were trapped within the tiny pores of the smaller pore size distribution in the Diamond structure, in this case resulting in a partially non-interconnected void, as shown in the enlarged view in Figure 4b. Those trapped powders were difficult to completely remove even by high pressure gas (0.6 MPa) and ultrasonic cleaning (for 20 min). They appeared in the height position of h ≥16 mm in the Diamond gradient structure, where the pore size of the CT model is 272 μm and the correspondingly designed pore size is 441 μm according to Table S3. While no clogging was observed in the Gyroid structure due to its minimum pore size of 283 μm, which is larger than 272 μm in Diamond.

As a result, in order to avoid the phenomenon of powder trapping, it is suggested that: when using SLM technology with processing conditions in this work, scaffolds should be designed to have a pore size of ≥ 450 μm for a good interconnectivity. Especially for a relative density of 30%, the size of the Gyroid porous unit should be ≥0.88 mm, and that of the Diamond should be ≥0.6 mm.

### 3.2. Mechanical Properties

#### 3.2.1. Stress–Strain Curves

Compressive tests were performed to investigate the mechanical behaviors of these cylindrical samples. Each set of samples was tested in triplicate, showing a highly reproducible pattern (Figures S1 and S2 in Supplememtary Materials). As stated by Wally et al. [41], it is the regular structures and reproducibility of SLM that leads to high repeatability for mechanical tests, which is superior to random structured porous materials such as foams and sponges. Consequently, due to the good manufacturing repeatability of SLM, it is beneficial to predict the mechanical properties of these regular porous structures.

**Figure 9:**
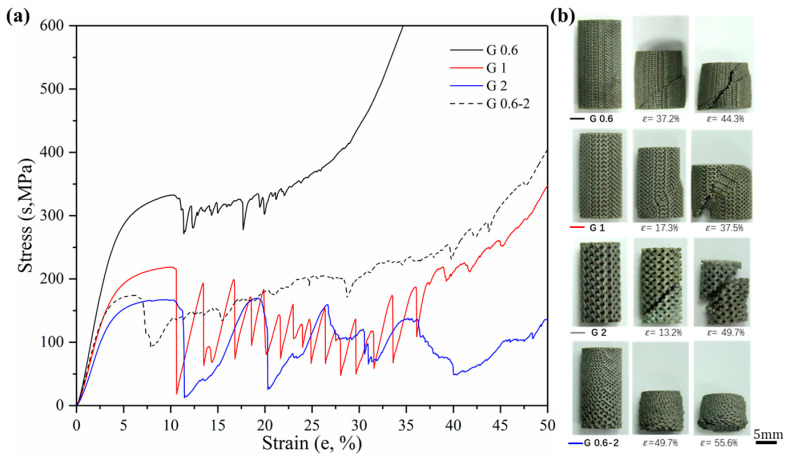
(**a**) Compressive stress-strain curves of Gyroid gradient in cell-size ranging from 0.6 to 2 mm, and (**b**) video frames at the corresponding strain from the compression.

**Figure 10:**
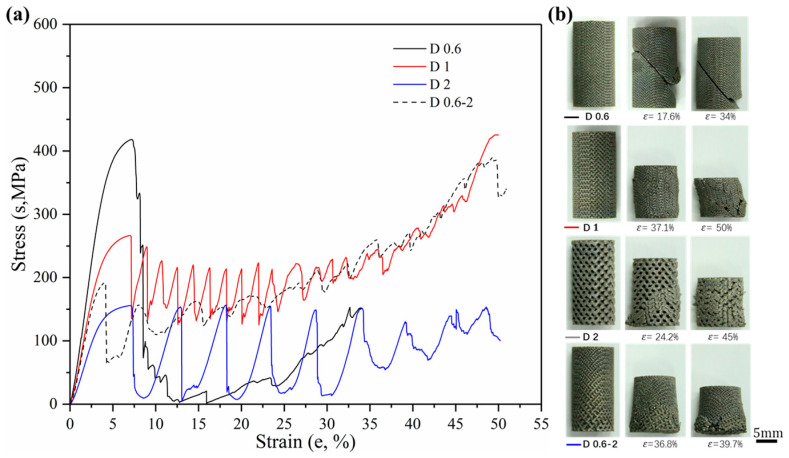
(**a**) Compressive stress-strain curves of Diamond gradient in cell-size ranging from 0.6 to 2 mm, and (**b**) video frames at the corresponding strain from the compression.

Sets of compressive stress–strain curves of Gyroid and Diamond structures are depicted in Figure 9 and Figure 10, respectively, along with their video frames under compression. In contrast, the uniform structures tend to present a severe drop in strength associated with shear collapse, and a smaller unit leads to a higher stress curve, while the gradient structure underwent sequential layer collapse from the bottom up, showing a significant plateau stress without a severe drop.

It is well known [33] that porous structures undergo four phases of elastic deformation, yield collapse, stress fluctuation (or plateau stress), and densification when subjected to compressive loads. For biological scaffolds, the elastic phase is of great concern, in which the elastic modulus needs to match that of human bone to reduce the stress shielding phenomenon of the implant [42]. A higher yield stress has a positive effect on improving the fatigue performance of implants [43].

According to the Gibson–Ashby model [9,44], the elastic modulus and yield stress can be tailored for load adaptation by adjusting the relative density of porous structures. That is, custom-designed mechanical properties have the potential to meet the actual demands of the implant site. However, these studies were based on porous structures with the same unit size and did not take into account the effects of pore size changes on mechanical properties. The study in this paper confirmed that a change in the cell size of the porous structure has a significant influence on its mechanical properties including the elastic modulus and yield strength, providing new considerations when designing scaffolds for load bearing.

#### 3.2.2. Strength and Modulus

**Figure 11:**
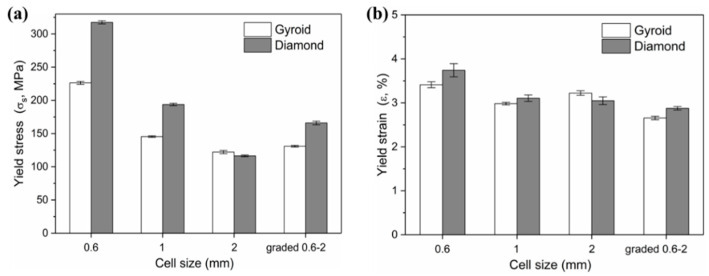
(**a**) Yield stress and (**b**) corresponding yield strain of Gyroid and Diamond scaffolds with cell size ranging from 0.6 to 2 mm.

Firstly, more attention is paid to the material properties before yielding in engineering practice, i.e., the yield strength and elastic modulus of the material. Figure 11 shows the yield strength and corresponding strain of Gyroid and Diamond scaffolds. Similarly, increasing cell size gradually reduces the yield strength. For the gradient structure, its strength is slightly higher than that of the structure with the maximum cell size. It is worth noting that the strength of D0.6 is abnormally high, which may be attributed to the effect of the trapped powder mentioned above on the strengthening of the mechanical properties. Interestingly, all of their yield strains remained consistent, regardless of the cell shape or size, indicating that the deformation of the two structures before yielding is relatively similar.

The elastic moduli, along with relevant yield strength and collapse strength, are listed in Table 2. The calculated elastic moduli for all porous structures were within the range for cancellous bone tissue (4 to 32 GPa [1,45]), which is a significant conclusion for the application of TPMS porous structures fabricated by SLM as artificial bone substitute material. As a consequence, the TPMS porous structures exhibit properties comparable with those of natural bone, which plays a crucial role in reducing the stress shielding effect and increasing the longevity of implants.

**Figure 12:**
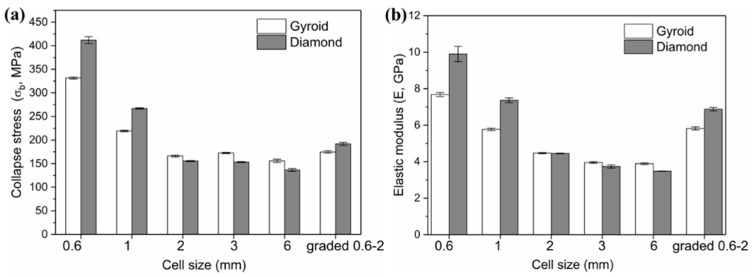
Comparison of (**a**) collapse stress and (**b**) elastic modulus of Gyroid and Diamond scaffolds with cell size ranging from 0.6 to 6 mm, where cell size of 3 and 6 mm are based on Reference [28].

In our previous article [28], identical structures with cell sizes of 3 and 6 mm, also covering the same relative density of 30%, were studied. Their properties were cited and listed in Table 2. And Figure 12 shows the collapse stress and elastic modulus of Gyroid and Diamond scaffolds with cell sizes ranging from 0.6 to 6 mm. It is worth noting the following:

For cell size C < 2 mm, the Diamond structure has collapse stress and elastic modulus superior to the Gyroid structure.

For C = 2 mm, the mechanical properties of both are substantially equivalent.

For 6 mm ≥ C ≥ 2 mm, the opposite situation occurs, leading to higher Gyroid properties. That is, the order of the mechanical properties of Gyroid and Diamond structures with different cell sizes is possibly ranked according to the cell size C = 2 mm.

Many experimental studies [9,26,46] have verified that Gyroid structures have superior mechanical properties, including strength, modulus of elasticity, and ductility. However, in these studies, the size of the porous unit is generally ≥4 mm, while for the small size range that is suitable for implants, there is little literature mentioning the difference in the performance between the Gyroid and Diamond structures. Here, an interesting phenomenon has been discovered, that is, when the cell size is <2 mm, the Diamond structure exhibits higher performances. This may be attributed to the increased material phenomenon analyzed earlier. Since the surface area of the Diamond structure is larger than that of the Gyroid under the same conditions, the amount of increased materials during the SLM process is proportional to the surface area of the part. Here it was found that the cell size offsetting the performance advantage of the Gyroid structure may be exactly C = 2 mm. Furthermore, Figure 5 shows that the fitting curve of the Diamond structure has a higher slope, contributing to a larger difference between the surface area of the Gyroid and Diamond structure as the cell size decreases, thus resulting in the significantly higher mechanical properties of the Diamond structure.

#### 3.2.3. Ductility

**Figure 13:**
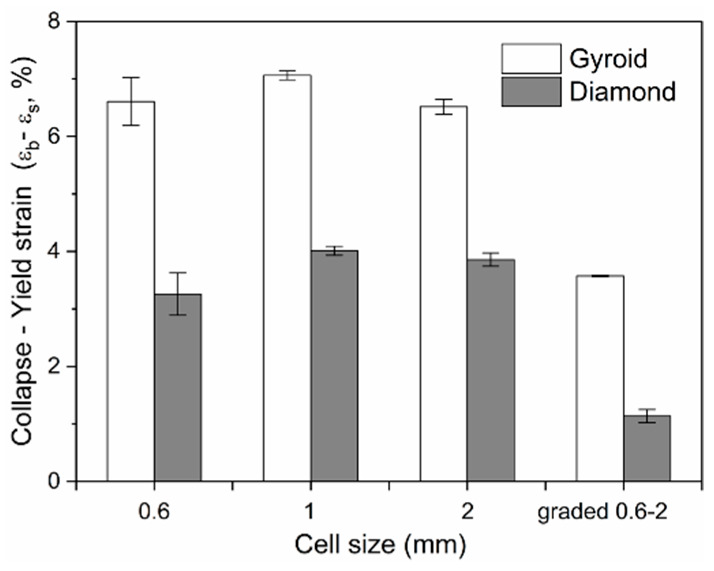
Comparison of the collapse–yield strain (*ε*_b_ − *ε*_s_) of Gyroid and Diamond scaffolds with cell sizes ranging from 0.6 to 2 mm.

The ductility is an important factor in the mechanical properties of porous structures, affecting the fatigue performance [47]. For an insight into understand their ductility, formula *ε*_b_ − *ε*_s_ was adopted to evaluate their bearing capacity after the first plastic failure, as shown in Figure 13, demonstrating that the value for each Gyroid unit size is significantly larger than that for Diamond. More precisely, the mean *ε*_b_ − *ε*_s_ value for Gyroid with the uniform structures of 0.6, 1, and 2 mm is 6.73% ± 0.29%, while that for Diamond counterparts is 3.71 ± 0.4%. The stress–strain curves in Figure 9 and Figure 10 also show that the collapse of the Diamond structure generally occurs at lower strain and is more abrupt than Gyroid. In addition, it can be seen from Figure 13 that their ductility is related to the unit shape, but independent on the unit size with no significant difference in the *ε*_b_ − *ε*_s_ value. However, the cell size gradient change adversely affects the collapse strain, and the *ε*_b_ − *ε*_s_ value dropped by 46.88% and 69.39% for Gyroid and Diamond, respectively (see Figure 13). In summary, the Gyroid has a better ductility than Diamond, and a change in cell size gradient reduces the ductility of the porous structure.

### 3.3. In Vitro Study

#### 3.3.1. Cell Adhesion

**Figure 14:**
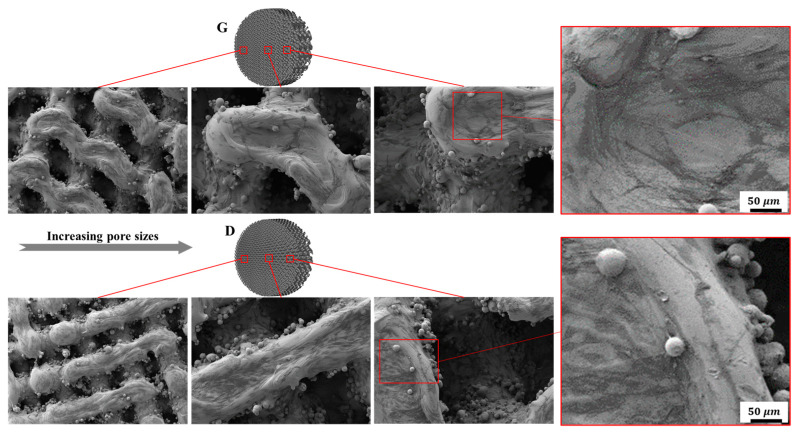
Representative SEM images of osteoblasts adhering to Gyroid and Diamond scaffolds with consecutive gradient in cell-sizes ranging from 0.6 to 2 mm.

After being cultured for 2 days, SEM was employed to observe cell adhesion on different substrates. It can be seen from Figure 14, that both the Gyroid and Diamond scaffolds display impressive cell attachment with increasing pore size. It is noteworthy that cells uniformly attach and spread on the surface of the scaffolds from the outside to inside, irrespective of the architecture and pore sizes of the scaffolds, due to their interconnected porosity inducing the attachment of bone cells. As is known, favorable cell adhesion is crucial for the survival of an implant [48], since it is beneficial to the subsequent biological events, for instance, cell spreading, proliferation, differentiation and eventually tissue formation [49,50]. The SEM images show that cells survived well on both Gyroid and Diamond scaffolds, indicating that the porous samples facilitate an ideal growth environment for osteoblast growth.

#### 3.3.2. Cell Morphology

**Figure 15:**
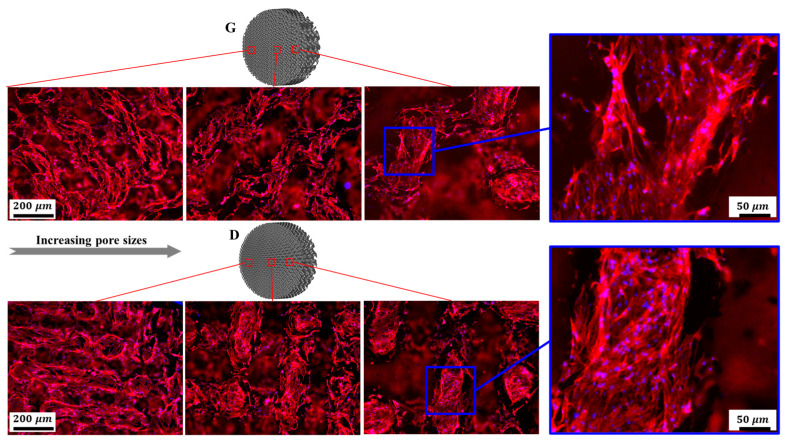
Representative fluorescence images of osteoblasts adhering to Gyroid and Diamond scaffolds with consecutive gradient in cell-sizes ranging from 0.6 to 2 mm. Cells were stained with actin filament (red), cell nuclei (blue).

Fluorescent microscopy was employed to further investigate cell morphology on the substrates. As shown in Figure 15, osteoblasts homogeneously spread along the struts of the scaffolds. It is worth mentioning that cells displayed elongated cytoskeletons with distinct spindly filopodia anchoring to the struts and oriented ordered arrangements along the frameworks of the scaffolds. Additionally, more cells adhered to the surfaces of the scaffolds with smaller pore sizes compared to the larger ones, which could be ascribed to the larger surface area and lower permeability coefficients [32]. Although a larger pore size provides ample space for nutrient–waste exchange and vascularization, the small pore porous structures with more surface area exhibit superior cell adhesion in the early stages of tissue growth. As mentioned in some reports [51,52], a higher mean curvature within a smaller porosity can induce more tissue proliferation in vitro. For the same structure with different pore sizes, the average curvature of the pores is inversely proportional to the pore size, and the smaller pore structure has a higher surface curvature, thus more cell adhesion occurs on the smaller pores of a stent. It was recognized in [32] that the higher cell seeding efficiency in the smaller pores is due to their lower permeability, which reduces the average fluid velocity and prolongs the stay time of the cell suspension within the scaffold, resulting in the facilitation of cell growth attached to the surface.

Nevertheless, it should be noted that fully interconnected pore architecture is an essential factor to facilitate tissue in-growth. And it was recommended that the pore size of scaffolds should be in a range of 300 to 900 μm, which is suitable for bone regeneration [3,4]. In the discussion of the pore distribution in the previous Section 3.1, the CT model showed that the pore size of the Gyroid scaffold varies from 283 to 1092 μm, and that of the Diamond scaffold ranges from 217 to 950 μm, which is associated with continuous gradient architecture similar to natural anatomical bone. The cells were homogeneously distributed in the gradient structure and showed metabolic activity similar to that of bone cells, which demonstrated that cells were evidently viable in the graded structures.

#### 3.3.3. Cell Viability and Differentiation

**Figure 16:**
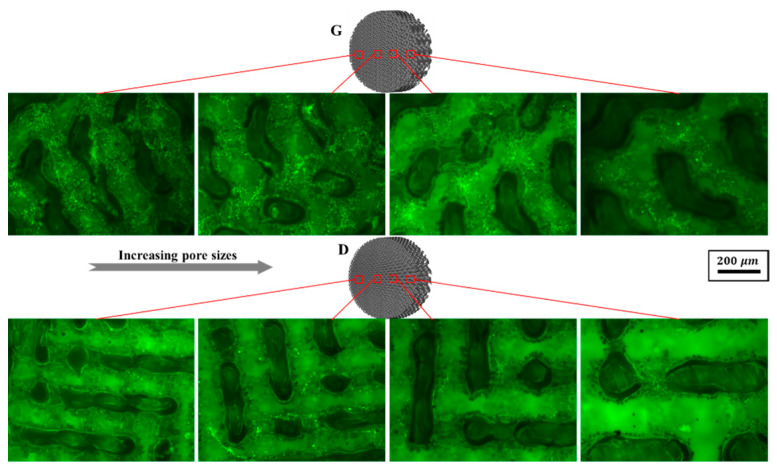
Representative fluorescein diacetate (FDA) staining fluorescence images of osteoblasts adhering to Gyroid and Diamond scaffolds with consecutive gradient in cell-sizes ranging from 0.6 to 2 mm, scale bar: 200 μm.

**Figure 17:**
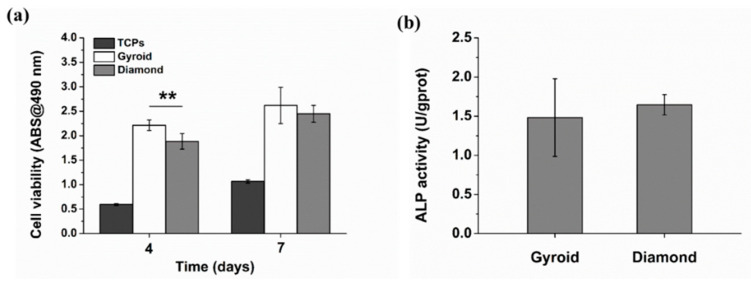
(**a**) Cell viability of osteoblasts based on MTT assay, (**b**) quantification of alkaline phosphatase (ALP) activity. Error bars represent means ±SD, ** *p* < 0.01, n = 5.

The cell viability was evaluated by FDA staining (Figure 16). Green fluorescence indicates living cells. It is noteworthy that osteoblasts covered on the surfaces of the scaffolds, while Gyroid scaffolds displayed higher cell density than the Diamond scaffolds. In addition, the cell viability was further quantified by MTT assay. With reference to Figure 17a, cells proliferated steadily over time; likewise, the cell viability on Gyroid scaffolds was higher than that on Diamond scaffolds at all times. Especially on the 4th day, there was a significantly higher cell viability (*p* < 0.01) on Gyroid scaffolds by contrast. The results indicated that scaffolds with gyroid cell structures were superior for osteoblast growth, which could be attributed to larger surface area for cell penetration and satisfactory medium fluid throughout the porous architectures. With respect to cell differentiation, ALP activity measurement after incubation for 7 days indicates there was no significant difference between the two scaffolds (Figure 17b).

Ran et al. [3] evaluated the biological response of the porous structure with straight circle channels, where it was found that cell viability was about 0.7 after 7 days of incubation. Additionally, osteointegration of four different pore structures were evaluated in the study reported by Wang et al. [53], and no significant difference among the four groups in cell morphology, viability and proliferation was found as well. Thanks to the inner smaller pores enabling cells to receive more biological stimulation and differentiate between the osteoblast and outer pores—giving larger open spaces for cells proliferation—ALP activity in scaffolds with pore size gradients was significantly greater than those in other groups at day 7. Liang et al. [54] reported that trabecular-like porous scaffolds with irregularity and higher porosity bring a decrease in mechanical properties, but enhance cells proliferation and osteoblast differentiation at an earlier time due to their preferable combination of small and large pores with various shapes. Thus scaffolds with graded pore size would be more suitable for cell sustaining viability and proliferation, owing to more cells seeding on small pores and the mass transport ability of larger pores.

## 4. Conclusions

In this study, we aimed to evaluate interconnected scaffolds with novel cell-size gradients in terms of their pore characteristics, mechanical properties and preliminary bio-functional response in vitro. Uniform and graded scaffolds with Gyroid and Diamond units were designed using the ISM method and fabricated by selective laser melting (SLM) with Ti6Al4V.

First, with micro-CT reconstruction, the morphological evaluation of these porous scaffolds gives a high repeatability of the SLM technology, showing continuous graded changes in pore size and surface area. However, due to the increased volume and rough surface of the SLM parts, the surface area of the gradient scaffolds increased by an average of 29.97% ± 5.1% mm^2^ and the pore size decreased by an average of 227 ± 46.1 μm compared with the design ones. As a result, it is recommended that the pore size of scaffolds should be designed to be ≥450 μm to ensure good interconnectivity.

Second, static mechanical tests reveal that both of Gyroid and Diamond have a superior strength (191.72 to 331.46 MPa) compared to natural bone, and their elastic moduli (4.45 to 9.9 GPa) are comparable with that of natural bone. In comparison, Gyroid exhibits better performance, including modulus of elasticity, ultimate strength and ductility. Unfortunately, graded change in cell size reduces the ductility for both Gyroid and Diamond scaffolds seriously compared to the uniform ones.

Finally, in vitro cell culture was performed on the gradient porous scaffold to evaluate the growth of bone cells within them. Benefitting from the biocompatibility, connectivity and suitable pore size of the porous Ti6Al4V material, the cells grow well and distribute uniformly inside the gradient porous samples, indicating that the gradients provide good cytocompatibility for osteoblast growth.

In future work, further experiments in vivo are needed to assess the biological response of porous gradients for clinical research. Furthermore, dynamic performance tests including impact and fatigue should be carried out to study the mechanical behavior of the porous structures.

## Figures and Tables

**Table 1:** Detailed information about the samples used for experiments.

Unit	Groups	Cell Size (mm)	Dimension (*ϕ* × h, mm)	Role
Gyroid	G 0.6	0.6	*ϕ*10 × 20	Mech-test
	G 1	1	*ϕ*10 × 20	Mech-test
	G 2	2	*ϕ*10 × 20	Mech-test
	G 0.6–2	Graded 0.6–2	*ϕ*10 × 20	Mech-test
	G vitro	Graded 0.6–2	*ϕ*15 × 5	Bio-test
Diamond	D 0.6	0.6	*ϕ*10 × 20	Mech-test
	D 1	1	*ϕ*10 × 20	Mech-test
	D 2	2	*ϕ*10 × 20	Mech-test
	D 0.6–2	Graded 0.6–2	*ϕ*10 × 20	Mech-test
	D vitro	Graded 0.6–2	*ϕ*15 × 5	Bio-test

**Table 2:** Designed characteristics and results of static compressive tests of cellular gradients in three patterns.

Cell Size (C, mm)		Elastic Modulus (E, GPa)	Yield Strength (*σ*_s_, MPa)	Collapse Strength (*σ*_b_, MPa)
0.6	G	7.68 ± 0.11	226.34 ± 2.18	331.46 ± 2.05
	D	9.90 ± 0.41	317.48 ± 2.34	411.71 ± 7.36
1	G	5.77 ± 0.07	145.45 ± 1.22	219.23 ± 1.62
	D	7.36 ± 0.13	193.82 ± 1.77	266.95 ± 1.42
2	G	4.47 ± 0.01	122.10 ± 2.40	166.14 ± 2.03
	D	4.45 ± 0.02	116.46 ± 1.30	155.49 ± 1.05
3 [28]	G	3.95 ± 0.05	135.55 ± 3.21	172.70 ± 1.46
	D	3.73 ± 0.09	136.11 ± 2.70	153.30 ± 1.09
6 [28]	G	3.89 ± 0.05	118.63 ± 1.32	155.84 ± 3.51
	D	3.48 ± 0.01	121.66 ± 2.20	136.65 ± 2.78
Graded 0.6–2	G	5.82 ± 0.08	130.95 ± 1.29	174.74 ± 2.72
	D	6.88 ± 0.09	166.03 ± 2.73	191.72 ± 3.38

## Supplementary Materials

The following are available online at https://www.mdpi.com/1996-1944/13/11/2589/s1, Figure S1: 1 Compressive stress-strain curve of Gyroid, Figure S2: Compressive stress-strain curve of Diamond, Table S1: Details of the selective laser melting process, Table S2: Comparison of physical data between designed and as-build models of Gyroid gradient porous structure, Table S3: Comparison of physical data between designed and as-build models of Diamond gradient porous structure.
