# Supplementary material for: Additively Manufactured Continuous Cell-Size Gradient Porous Scaffolds: Pore Characteristics, Mechanical Properties and Biological Responses In Vitro
Source: Materials (Basel). 2020 Jun 5;13(11):2589. doi: 10.3390/ma13112589 (PMC7321598; doi:10.3390/ma13112589)
Supplement: Supplementary file 1 [file materials-13-02589-s001.pdf]

Table S1. Details of the selective laser melting process.

laser power W	spot size μm	layer thickness μm	scanning speed mm/s	hatching space mm	O_2 content vol.%
175	100	30	1250	0.1	≤ 0.1

In addition, a raster scanning strategy was employed with a 67° rotation of scanning direction between two layers. The build platform was held at 80 °C during specimen production.

Main Program:

```
clear;
d=0.1; xa=-5*pi; xb=5*pi; ya=-5*pi; yb=5*pi; za=0; zb=10*2*pi;      %units=5*5*10
[x,y,z] = meshgrid(xa:d:xb, ya:d:yb, za:d:zb);
m=3;                  % cell size graded coefficient. m=1 indicates a uniform structure.
K1=(m-1)/(zb-za); C1=-za*K1+1; C0=1/2*K1*za^2;
a=@(x,y,z) K1*z+C1; b=@(x,y,z) K1*z+C1; c=@(x,y,z) K1/2*z+C1+C0/z;
fun=G(x,y,z);   or   fun=D(x,y,z)                    %calling Program 2 or 3
v=fun(x,y,z); fv=isosurface(x,y,z,v,0);   h = patch(fv);
set(h,'FaceColor','g','EdgeColor','none');
xlabel('x');ylabel('y');zlabel('z'); grid on; view([1,1,1]); axis equal; camlight; lighting gouraud;
hold on;
```

Program2:

```
G=@(x,y,z)...
      cos(a(x,y,z).*(x-1/4*pi)).*sin(b(x,y,z).*(y-1/4*pi))...
     +cos(b(x,y,z).*(y-1/4*pi)).*sin(c(x,y,z).*(z-1/4*pi))...
     +cos(c(x,y,z).*(z-1/4*pi)).*sin(a(x,y,z).*(x-1/4*pi))...
     +0.08*(cos(2*a(x,y,z).*(x-1/4*pi)).*cos(2*b(x,y,z).*(y-1/4*pi))...
     +cos(2*b(x,y,z).*(y-1/4*pi)).*cos(2*c(x,y,z).*(z-1/4*pi))...
     +cos(2*c(x,y,z).*(z-1/4*pi)).*cos(2*a(x,y,z).*(x-1/4*pi)))+0.60559;     % relative density = 30%
```

Program 3:

```
D=@(x,y,z)...
   sin(a(x,y,z).*(x-1/4*pi)).*sin(b(x,y,z).*(y-1/4*pi)).*sin(c(x,y,z).*(z-1/4*pi))...
   +sin(a(x,y,z).*(x-1/4*pi)).*cos(b(x,y,z).*(y-1/4*pi)).*cos(c(x,y,z).*(z-1/4*pi))...
   +cos(a(x,y,z).*(x-1/4*pi)).*sin(b(x,y,z).*(y-1/4*pi)).*cos(c(x,y,z).*(z-1/4*pi))...
   +cos(a(x,y,z).*(x-1/4*pi)).*cos(b(x,y,z).*(y-1/4*pi)).*sin(c(x,y,z).*(z-1/4*pi))...
    -0.07.*(cos(4.*a(x,y,z).*(x-1/4*pi))…
        +cos(4.*b(x,y,z).*(y-1/4*pi))+cos(4.*c(x,y,z).*(z-1/4*pi)))+0.48892;     % relative density = 30%
```

Table S2. Comparison of physical data between designed and as-build models of Gyroid gradient porous structure.

Layer	Designed model			As-build model (CT)				
	Surface (S_0, mm^2)	Volume (V_0, mm^3)	Pore size (P_0, mm)	Surface (S_1, mm^2)	Volume (V_1, mm^3)	Pore size (P_1, mm)	Relative density (ρ*, %)	$\delta = \Delta V/S_0$ (mm)
1	129.550	22.996	1.373	160.321	29.474	1.092	37.527	0.050
2	140.121	22.646	1.285	168.61	28.677	0.998	36.513	0.043
3	148.138	22.704	1.158	174.064	29.678	0.873	37.787	0.047
4	159.778	23.152	1.095	196.233	30.06	0.838	38.274	0.043
5	170.351	22.818	1.007	206.317	30.599	0.792	38.960	0.046
6	182.135	23.037	0.954	225.601	32.241	0.702	41.051	0.051
7	191.367	22.573	0.878	235.166	32.963	0.673	41.970	0.054
8	203.395	22.784	0.815	255.236	34.602	0.583	44.057	0.058
9	212.232	22.929	0.802	267.538	35.579	0.567	45.301	0.060
10	222.626	22.776	0.765	285.436	36.982	0.513	47.087	0.064
11	234.727	22.818	0.729	295.638	38.435	0.453	48.937	0.067
12	244.092	22.84	0.702	306.604	40.118	0.430	51.080	0.071
13	255.575	22.673	0.650	319.131	41.642	0.433	53.020	0.074
14	265.388	22.731	0.641	332.064	43.003	0.400	54.753	0.076
15	275.435	22.675	0.587	344.082	45.149	0.372	57.485	0.082
16	287.559	22.804	0.575	344.992	46.421	0.353	59.105	0.082
17	296.733	22.709	0.549	359.888	48.28	0.328	61.472	0.086
18	308.995	22.639	0.534	367.933	50.297	0.315	64.040	0.090
19	318.724	22.863	0.525	370.183	52.273	0.322	66.556	0.092
20	329.502	22.563	0.511	373.547	52.722	0.283	67.128	0.092

Table S3. Comparison of physical data between designed and as-build models of Diamond gradient porous structure.

Layer	Designed model			As-build model (CT)				
	Surface (S_0, mm^2)	Volume (V_0, mm^3)	Pore size (P_0, mm)	Surface (S_1, mm^2)	Volume (V_1, mm^3)	Pore size (P_1, mm)	Relative density (ρ*, %)	$\delta = \Delta V/S_0$ (mm)
1	157.835	23.428	1.090	198.62	32.353	0.950	41.193	0.057
2	175.118	22.992	0.994	211.491	32.203	0.793	41.002	0.053
3	181.293	22.121	0.912	222.498	33.865	0.658	43.118	0.065
4	196.122	23.267	0.804	250.241	34.903	0.635	44.440	0.059
5	212.132	23.217	0.762	266.626	36.868	0.563	46.942	0.064
6	222.462	22.707	0.741	287.615	38.329	0.502	48.802	0.070
7	234.728	22.745	0.665	305.546	39.668	0.470	50.507	0.072
8	248.496	22.453	0.627	326.64	41.603	0.478	52.971	0.077
9	263.969	23.221	0.640	360.236	43.133	0.385	54.919	0.075
10	272.855	22.802	0.659	374.881	44.076	0.333	56.119	0.078
11	288.49	22.756	0.625	388.068	44.143	0.310	56.205	0.074
12	302.107	22.817	0.587	399.368	43.753	0.300	55.708	0.069
13	314.43	22.567	0.550	408.006	44.515	0.323	56.678	0.070
14	327.686	22.666	0.534	428.357	43.92	0.275	55.921	0.065
15	339.305	22.599	0.452	433.947	45.26	0.308	57.627	0.067
16	355.989	22.755	0.441	462.488	47.218	0.272	60.120	0.069
17	366.853	22.648	0.419	509.683	49.701	0.255	63.281	0.074
18	382.478	22.554	0.392	519.077	51.075	0.245	65.031	0.075
19	395.607	22.853	0.433	517.113	51.426	0.217	65.478	0.072
20	405.586	22.34	0.425	492.869	50.57	0.217	64.388	0.070

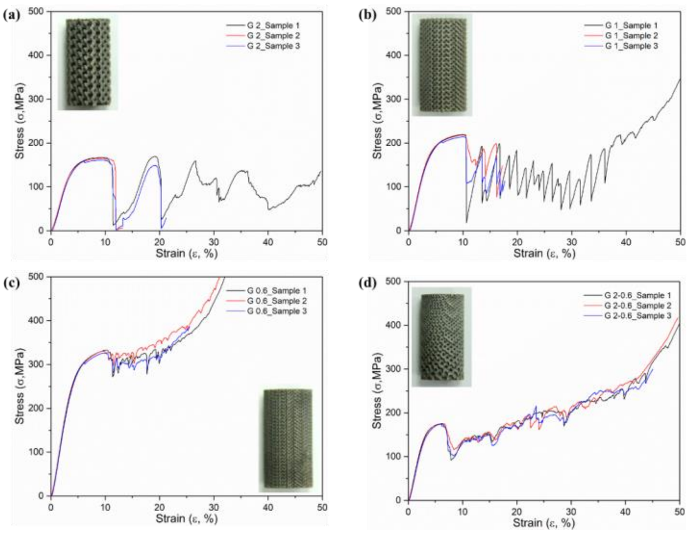

Figure S1. Compressive stress-strain curve of Gyroid, (**a**) cell size 2 mm, (**b**) cell size 1 mm, (**c**) cell size 0.6 mm, (**d**) graded cell size 2–0.6 mm, showing a highly reproducible pattern.

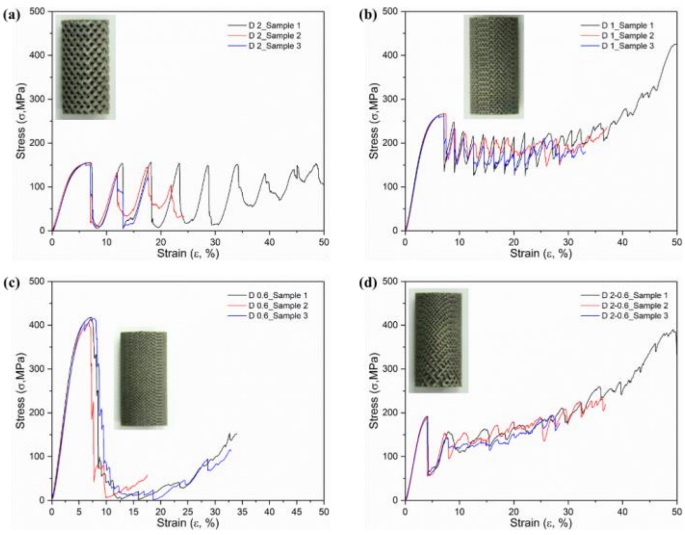

Figure S2. Compressive stress-strain curve of Diamond, (**a**) cell size 2 mm, (**b**) cell size 1 mm, (**c**) cell size 0.6 mm, (**d**) graded cell size 2–0.6 mm, showing a highly reproducible pattern.
